# Using Graphene Liquid Cell Transmission Electron Microscopy to Study *in Situ* Nanocrystal Etching

**DOI:** 10.3791/57665

**Published:** 2018-05-17

**Authors:** Matthew R. Hauwiller, Justin C. Ondry, A. Paul Alivisatos

**Affiliations:** ^1^Department of Chemistry, University of California-Berkeley; ^2^Department of Material Science and Engineering, University of California-Berkeley; ^3^Kavli Energy NanoScience Institute; ^4^Materials Sciences Division, Lawrence Berkeley National Laboratory

**Keywords:** Chemistry, Issue 135, Graphene Liquid Cell, Transmission Electron Microscopy, *in situ *Transmission Electron Microscopy, nanocrystals, oxidative etching, gold nanorods, single nanoparticle experiments

## Abstract

Graphene liquid cell electron microscopy provides the ability to observe nanoscale chemical transformations and dynamics as the reactions are occurring in liquid environments. This manuscript describes the process for making graphene liquid cells through the example of graphene liquid cell transmission electron microscopy (TEM) experiments of gold nanocrystal etching. The protocol for making graphene liquid cells involves coating gold, holey-carbon TEM grids with chemical vapor deposition graphene and then using those graphene-coated grids to encapsulate liquid between two graphene surfaces. These pockets of liquid, with the nanomaterial of interest, are imaged in the electron microscope to see the dynamics of the nanoscale process, in this case the oxidative etching of gold nanorods. By controlling the electron beam dose rate, which modulates the etching species in the liquid cell, the underlying mechanisms of how atoms are removed from nanocrystals to form different facets and shapes can be better understood. Graphene liquid cell TEM has the advantages of high spatial resolution, compatibility with traditional TEM holders, and low start-up costs for research groups. Current limitations include delicate sample preparation, lack of flow capability, and reliance on electron beam-generated radiolysis products to induce reactions. With further development and control, graphene liquid cell may become a ubiquitous technique in nanomaterials and biology, and is already being used to study mechanisms governing growth, etching, and self-assembly processes of nanomaterials in liquid on the single particle level.

**Figure:**
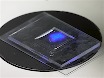


## Introduction

Controllably synthesizing nanocrystals^1^ and assembling nanoparticles into larger structures^2^,^3^ requires an understanding of the fundamental mechanisms governing how atoms and nanoparticles interact and bind together. Ideally, studies of these nanoscale processes would be performed in their native liquid environment with the corresponding spatial resolution necessary to observe the phenomena of interest, but these requirements pose challenges due to the nanometer length scale on which these systems operate. Researchers have long desired to use the spatial resolution of electron microscopy to image these processes, but the high vacuum of the electron microscope column requires encapsulation of the liquid solution^4^. Some early liquid cell electron microscope experiments encapsulated liquid between two silicon nitride membranes^5^,^6^,^7^,^8^, and this method has now become a commercially available technique for studying dynamic nanoscale processes.

Commercially available silicon nitride liquid cell TEM holders have provided the necessary resolution to see and understand a variety of interesting phenomena on the nanoscale^9^,^10^,^11^,^12^,^13^,^14^,^15^,^16^. Some commercial liquid cell TEM holders have additional capabilities such as heating, flow, and electrical connections that further expand the realm of nanoscale processes that can be investigated. However, with all of these capabilities, the commercial systems are not optimized around achieving the highest spatial resolution. For researchers that need improved spatial resolution, decreasing the window thickness and decreasing the liquid thickness are two potential routes to less electron beam scattering and better resolution^17^. Some groups who use silicon nitride liquid cells fabricate their own windows which yields greater control over the window and liquid thicknesses.^18^ The decreased scattering of these home-made liquid cells has enabled electron microscopy studies with greater spatial resolution including atomic resolution studies^19^,^20^,^21^.

Since the thickness of the encapsulating material is one aspect that negatively affects the spatial resolution of the liquid cell experiments, atomically thin, low-Z materials such as graphene would be ideal encapsulating materials^22^,^23^. Graphene sheets are still strong enough to protect the liquid pockets from the pressure difference of the column. In addition, these graphene liquid cell pockets usually contain thinner layers of liquid, further enhancing the achievable spatial resolution. Many interesting nanoscale processes have been investigated with graphene liquid cells including studies following nanoparticle facet trajectories and nanoparticle dynamics with atomic resolution^23^,^24^,^25^,^26^,^27^. An unintended advantage of the graphene liquid cell technique is that this high spatial resolution can be achieved without requiring the purchase of a different TEM holder or specialized silicon fabrication. Experiments using silicon nitride cells that achieved high resolution also required large nanoparticles composed of heavy atoms, whereas the resolution gained by the graphene liquid cell can provide atomic resolution for sub-2 nm nanoparticles^25^. Additionally, the graphene liquid cell has opened opportunities for studying biological samples with electron microscopy due to the flexible nature of graphene for encapsulation^28^,^29^ and the ability of graphene to mitigate some of the damaging effects of the electron beam^30^. Due to these advantages, graphene liquid cell electron microscopy has the potential to become a standard technique in the nanoscience community once greater numbers of researchers understand better whether this technique can help their research and how to apply this technique.

Researchers in chemical, nanomaterial, biological, and other fields desiring spatial resolution of *in situ* transformations can benefit from employing graphene liquid cell electron microscopy technique. This* in situ* method is especially valuable for non-equilibrium processes that require visualization during the transformation. One significant drawback of liquid cell TEM techniques is the generation of radiolysis species by the perturbative electron beam^31^, which can induce undesirable changes in delicate samples. Researchers have developed models to try to quantify the beam-driven chemistry^31^,^32^, and strategies are being developed to mitigate these effects^30^,^32^. Graphene liquid cell TEM has the additional challenge of being fragile and often difficult to make, especially for researchers new to the technique. The aim of this article is to share the details of how graphene liquid cell TEM experiments can be carried out (**Figure 1**), using an example experiment observing single particle etching of nanocrystals, and hopefully show that graphene liquid cell experiments are possible for almost any group with access to an electron microscope. The protocol will cover graphene coating of grids, liquid cell formation, TEM use for graphene liquid cell etching experiments, and image analysis techniques. Critical steps in making the liquid cells such as the size of the droplet encapsulated, careful consideration of liquid solution contents, and use of only direct transfer graphene will be covered with additional advice on how to avoid repeating the pitfalls of previous researchers. Graphene liquid cell TEM is an emerging technique for nanoscale research, and this article will enable new entrants to begin utilizing this technique.

## Protocol

### 1. Making Graphene-Coated TEM Grids

1. Cut out a roughly 2 cm^2^ piece of premade graphene-on-copper (see **Table of Materials**) which fits around 6 to 8 TEM grids. NOTE: Using 3 - 5-layer graphene instead of single layer graphene encapsulates liquid pockets with higher success rates without losing resolution. Since graphene is an atomically thin, low-Z material, most resolution loss is from liquid thickness for graphene liquid cells.
2. Clean the graphene using an acetone wash (Figure 2A). NOTE: This step is designed to remove any residual PMMA [poly(methyl methacrylate)] left on the graphene surface during the deposition process. If the user is confident that their graphene is clean, this step is unnecessary.
  1. Place the graphene-on-copper piece in a glass Petri dish and fill with acetone. NOTE: Acetone is used because PMMA dissolves in acetone.
  2. Gently heat the acetone solution (~50 °C) for 5 min, swirling the solution periodically. NOTE: Make sure to watch the acetone and temperature to avoid a fire. This should be done in a fume hood.
  3. Remove the graphene-on-copper piece from the acetone wash with tweezers and replace the acetone with new, clean acetone. NOTE: Be careful not to scrape or otherwise damage the graphene surface with the tweezers.
  4. Repeat the washing process a total of 3 times.
  5. Let the graphene-on-copper air-dry thoroughly before going on to the next step.
3. Smooth out the graphene-on-copper piece to remove any macroscopic wrinkles (Figure 2B). NOTE: This smoothing process is performed to ensure that the perforated support foils-TEM grids (see the **Table of Materials**) are able to properly bond to the graphene surface. Bumps and creases in the graphene-on-copper make it difficult to maintain good contact.
  1. Take two clean glass slides and place a folded wipe (see **Table of Materials**) on the bottom glass slide. On top of the wipe, place the graphene-on-copper piece. Finally, place the second glass slide on top. NOTE: Place the graphene-on-copper piece with the graphene side up (touching glass slide) to prevent scratching from the tissue wipe. The folded tissue is used to gradually push out the wrinkles and prevent folding in new creases.
  2. Press down on the top slide, gradually smoothing out any wrinkles in the graphene-on-copper piece. Reduce the number of folds in the tissue and repeat the pressing process. Continue the process until a final pressing between the two glass slides with no tissue wipe.
4. Lay down TEM grids on the graphene-on-copper piece (Figure 2C).
  1. Place the holey amorphous carbon support foil-TEM grids (see **Table of Materials**) down on the graphene with the amorphous carbon in contact with the graphene. NOTE: Be careful not to bend or deform TEM grids when picking them up with the tweezers. Bent TEM grids do not bind properly to the graphene. Picking the grids up by the edge of the grid prevents deformation of the grids. Here, gold TEM grids are used to avoid etching the grids during the step that removes the copper from the graphene-on-copper.
  2. Place a couple droplets of isopropanol on the grids. NOTE: If any grids become overlapped, gently move them with the tip of a tweezers after putting isopropanol on the grids. Be careful not to damage the graphene surface.
  3. Let dry for 2+ h to make sure grids are properly bonded. This drying process brings the holey amorphous carbon into better contact with the graphene. NOTE: To check whether the grids have adhered to the graphene, gently pick up the piece of graphene-on-copper and turn it upside down. If gravity does not remove the grids, they should be properly bonded.
5. Etch the copper using a sodium persulfate solution (Figure 2D).
  1. Make a solution with 1 g of sodium persulfate in 10 mL of deionized water.
  2. Using tweezers, carefully place the graphene-on-copper piece on the sodium persulfate solution with the copper side down. Let the piece float on the top of the sodium persulfate solution (Figure 2D).
  3. Keep the solution with graphene-coated grids sitting overnight. Note that the solution will become blue as the copper etches, and there will be no visible copper behind the graphene sheet when etching has finished (Figure 2E).
6. Wash the grids to clean off the sodium persulfate.
  1. Remove the floating grids from the solution and place them on top of clean, deionized water (see **Table of Materials** for filter) in a second Petri dish. NOTE: The easiest method to transfer the grids involves using a glass slide to pick up the grids and then placing them down in the second Petri dish filled with water. Some grids will fall to the bottom of the Petri dish during the transfer process. This is usually a sign that the graphene on the grid is cracked or otherwise damaged.
  2. Repeat this process 3 times to remove all sodium persulfate residue from the graphene-coated grids.
  3. Pick up the grids with tweezers, place the grids graphene-side up on a filter paper, and let them dry. NOTE: This final transfer out of the water wash can be difficult, as the grids often stick to the tweezers due to the capillary forces from residual water.

### 2. Making Liquid Cell Pockets

1. Take two graphene-coated TEM grids and place them graphene side up on a glass slide. Using a small surgical scalpel blade, cut off the edge of one of the graphene-coated TEM grids, approximately 1/4 to 1/8 of the area of the grid (Figure 3A). NOTE: Cutting one of the grids is hypothesized to bring the graphene on the two grids in closer contact to provide better graphene-graphene interaction to form pockets.
2. Prepare the solution to be encapsulated. NOTE: The solution is specific to the nanocrystal etching experiment.
  1. Make Tris buffer HCl solution with deionized water at a concentration between 10-100 mM. NOTE: We have found that for preparing aqueous metallic nanoparticle solutions, Tris buffer HCl leads to a higher success rate of stable pockets although more studies are needed to understand why Tris buffer HCl helps make stable pockets. Using Tris buffer base or no Tris buffer both seem to have much lower success rates of pocket formation in this case. Every solvent and sample likely will require optimization to find conditions which create stable pockets while not disrupting the chemistry being studied. A brief survey of the literature shows success with ortho-dichlorobenzene/oleylamine (9:1 ratio),^23^ 0.5x Tris-borate-EDTA (TBE) and 200 mM NaCl solution,^33^ and aqueous 0.15 M NaCl solution^30^ as well as the aqueous Tris buffer HCl system presented here.
  2. Make a 40 mM FeCl_3_ solution in a solution of deionized water with 1.8 µL of HCl per mL water. NOTE: The FeCl_3_ is the etchant for this etching experiment. Other experiments may add different solutions depending on the experiment being performed.
  3. Make gold nanorods and concentrate the nanorod sample after cleaning^1^,^34^.
  4. Mix 0.15 mL of 0.01-0.1 mM Tris Buffer HCl, 0.1 mL of 40 mM FeCl_3_ in HCl, and 10 µL of nanorods.
3. Place ~0.5 µL droplet of solution to be encapsulated on the non-cut graphene-coated TEM grid. Use a tweezers to hold the edge of the TEM grid down while placing the droplet so that the capillary forces do not pick up the TEM grid (Figure 3B). NOTE: Be careful to make the droplet as small as possible and place it as close to the center of the grid as possible.
4. Quickly and carefully place the graphene-coated TEM grid with the cut corner on top of the droplet; the goal is to have the second grid come to rest on top of the first grid with no liquid getting squeezed out (Figure 3C). NOTE: Having the second grid already placed in self-closing tweezers can make this process quicker and easier. This is arguably the trickiest step of the liquid cell formation process with many potential failures that can occur. Setting the top grid down while removing the tweezers is a challenge as the tweezers can get stuck in between the two grids. Generally, placing one edge of the top TEM grid down and then gradually letting go of the grid works best. Note that if liquid is seen on the glass slide, then the pockets probably did not seal properly.
5. Wait 5 min to let graphene liquid cell pockets form. NOTE: Some evaporation of the liquid may occur as the pockets are forming, but once a hermetic seal is formed, no additional liquid loss is likely. The relative concentrations of each species in solution should remain constant.
6. Bring the sample to the TEM for imaging. NOTE: The amount of time set aside for sealing varies from researcher to researcher. For etching experiments, less time before bringing the liquid cell to the TEM is desirable to avoid pre-etching.

### 3. Loading and Imaging Graphene Liquid Cell

Note:The operation of the Transmission Electron Microscope followed standard procedures found in the user manual. Every TEM will have different alignment procedures.

1. Place the graphene liquid cell in a traditional TEM single tilt holder (Figure 4). NOTE: Other standard holders such as double tilt holders or heating holders can be used as well. Holders that use a screw-like mechanism to secure the TEM grid may impose a shear force that destroys the graphene liquid cell.
2. Load the TEM holder into the TEM column. NOTE: Since the graphene liquid cell contains such a small volume of liquid with no reservoir and has separate pockets, there is no need to rigorously check for leaks like silicon nitride liquid cell experiments. Even if a graphene liquid cell pocket burst, only a very small amount of liquid is released and thus would not crash the TEM vacuum system.
3. Use the nanoparticles and amorphous carbon in the sample to properly align the TEM beam (gun tilt, condenser aperture alignment, and condenser stigmation), and image (Z-height adjustment, objective stigmation, rotation center alignment, and aberration corrector tuning if applicable). Then remove the holder from the beam path and calibrate electron beam dose rate.
  1. Turn the TEM filament on at least 20 min prior to calibration to allow it to stabilize for reproducible dose rates; this waiting time may be different depending on the TEM system and electron gun type. NOTE: Election microscopists often use dose rate to refer to the number of electrons delivered per unit area per unit time (e^-^/Å^2^s). In the radiation chemistry community, this is known as the flux density, and dose rate is defined as the amount of energy absorbed per unit area per unit time. Since calculating the amount of energy absorbed by a sample is difficult for complex geometries found in liquid cells, and to maintain consistency with the TEM community, we choose to use dose rate to refer to electrons per unit area per unit time.
  2. Condense the beam to the most condensed amount, highest dose rate, needed for the experiment using the viewing screen (Figure 5A). Read out and save lens current for the condensed beam. NOTE: For steps 3.3.2 to 3.3.5, a custom digital micrograph script was written which takes control of the condenser system of the TEM to calibrate the second condenser (C2) lens current with the delivered electron dose rate. This allows the researcher to reproducibly set the electron dose rate to arbitrary values during the experiment.
  3. Spread the beam to the most spread amount, lowest dose rate, needed for the experiment using the viewing screen (Figure 5B). Read out and save the lens current for the spread beam.
  4. Divide the range of condenser lens currents into 10 equally spaced values and collect images for each condenser lens value with the CCD camera.
  5. Convert CCD counts to dose rate using CCD sensitivity and magnification calibration for each lens current.
  6. Use data of electron flux at different lens currents to make a calibration curve. Use this calibration curve for the rest of the experiment to control the electron beam to the desired flux.
  7. Re-insert the sample to the beam path.
4. Begin searching for nanoparticles in liquid pockets while keeping the dose rate low (usually around 20 e^-^/Å^2^s). NOTE: Keeping the dose rate low prevents the nanoparticles from etching while searching for nanoparticles.
5. When a nanoparticle is found in a liquid pocket, fine tune the focus on the nanoparticle while maintaining a low dose rate. NOTE: Determining whether a nanoparticle is in a liquid pocket can be tricky, but the presence of bubbles or movement of particles is often a good sign of a stable liquid pocket. Sometimes, instead of liquid, the pockets resemble a very dense gel with bubbles moving extremely slowly. These situations are caused by evaporation of the liquid potentially due to unsealed pockets or cracks in the graphene. It is fairly easy to distinguish between gels with no movement and liquid environments with bubbles rapidly moving and changing shape. There may be some evaporation during the formation of good liquid cell pockets, but relative concentrations between reactants stays constant.
6. Use the calibration curve (see step 3.3 for this) to set the condenser lens current for the desired dose rate (Figure 5D). NOTE: An in-house script is used to set condenser lens current and image acquisition parameters
7. Begin collecting a time series of TEM images with metadata of dose rate and time stamps embedded in the image file.
8. After the particle has finished etching, spread the beam and begin looking for other nanoparticles in the liquid pockets.
9. When a sufficient amount of nanoparticle etching videos have been collected, remove the TEM holder from the TEM following standard TEM procedures. Take the graphene liquid cell out of TEM holder. NOTE: A typical imaging session lasts around 2-3 h with approximately 30 videos taken. The number of videos with usable data depends on the quality of the pockets and type of etching experiment.

### 4. Image Analysis of TEM Videos Using Computational Software

Note: Since TEM videos are 2-dimensional projections of 3-dimensional shapes, careful image analysis needs to be done to extract etching rates or shape changes.

1. Convert the native DM3 video files to an avi format using ImageJ and import the avi videos into computational software (see **Table of Materials**).
2. Analyze each nanorod in each frame of the video.
  1. Determine the outline of the nanorod by thresholding the image (Figure 7A). NOTE: The high contrast of the metallic nanoparticles makes image analysis easier. For studying systems with lower contrast, additional filters may be needed before thresholding.
  2. From the outline of the nanorod, determine the major and minor axis of the closest fit ellipse (Figure 7B). NOTE: The built-in image analysis software to determine the major and minor axis assumes the shape is an ellipse. For a nanorod, which is not an ellipse, these values should not be used when sizing nanoparticles.
  3. Use the major axis to cut the nanorod outline into two halves (Figure 7C).
  4. With each of these halves, determine the volume and surface area of the shape encompassed by rotating that outline around the major axis. NOTE: This calculus method is sometimes referred to as the method of rings. This method of analysis only works if the nanorod is symmetric around the major axis. Having two halves to compare volumes and surface areas provides some reassurance that the nanorod is truly rotational symmetric.
3. After extracting the volume and surface area of the nanorod for each frame of the video, compile and interpret the data. NOTE: This outlining method also allows for analysis of the facets of nanocrystals with defined shapes.

## Representative Results

Frames from a representative video of a nanorod etching under an electron beam dose rate of 800 e^-^/Å^2^s are shown in Figure 6. The solution requires about 20 s of beam illumination before the nanorod begins undergoing oxidative etching. After the nanorod begins etching, the rate of removal of atoms stays steady while the nanorod also maintains a constant aspect ratio. The nanorods typically do not have significant movement during the videos which is consistent with previous liquid cell TEM work using nanoparticles of this size^24^. Since the nanoparticles do not move much, bubble generation and bubble movement are usually the best ways to determine whether a nanoparticle is in a liquid pocket. As the nanorod becomes small, the nanorod begins rotating and moving in and out of the focal plane, confirming that the nanorod is in a liquid environment.

The most common failure of graphene liquid cells is the inability to encapsulate stable pockets of liquid. Sometimes this can lead to completely dry pockets characterized by no bubbles and no nanoparticle movement or size change. Additionally, a pocket can begin with liquid and bubbles but later dry out before the nanoparticle completely etches. Usually for a good liquid cell, each pocket is stable for around 2-3 min at the etching dose rate, and pocket drying only becomes a problem for large nanoparticles or slow etching processes. Sometimes, liquid can evaporate out of a pocket and leave behind a gel-like solution with a very high salt concentration. These gels are usually readily apparent when imaging due to the high contrast of the solution and extremely slow movement of bubbles and particles. Data collected in these gel-like solutions cannot be trusted.

After collecting the liquid cell TEM data, the videos with nanoparticle etching are analyzed. The volumes, surface areas, and facets (if applicable) can be extracted and evaluated further (Figure 7). One indication of a drying pocket is substantial slowing down of the rate of etching over time, so plotting the volume against time can be an effective method for checking the stability of the pocket and reliability of the data. Other suboptimal results include non-symmetric etching indicative of inhomogeneous pocket contents and undesirable precipitation of iron hydroxide species from the iron chloride etchant. Overall, the most important key for successful graphene liquid cells is a stable liquid environment that leads to reproducible nanocrystal dynamics over multiple nanoparticles and liquid pockets.

**Figure:**
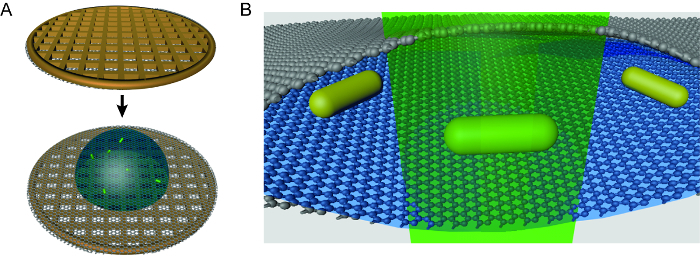

Figure 1**. Schematic of graphene liquid cell TEM technique. (A)** To assemble a graphene liquid cell, a droplet of solution is placed on a graphene-coated holey carbon TEM grid. A second graphene-coated grid is placed on top of the droplet to form a pocket. Note that this image is not to scale and the liquid droplet is about 33% too large. **(B)** Zoomed-in schematic of a liquid pocket during TEM imaging of gold nanorods. This cartoon is also not to scale. Please click here to view a larger version of this figure.

**Figure:**
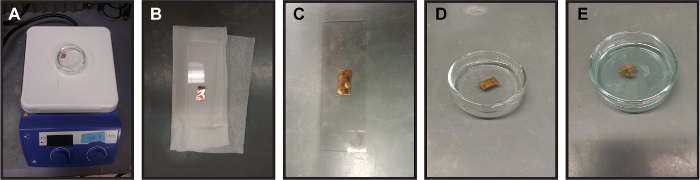

Figure 2**. Process for making graphene coated TEM grids (A)** Washing the graphene-on-copper piece in warm acetone **(B)** Removing macroscopic wrinkles by flattening graphene-on-copper between two glass slides. A tissue is placed beneath the graphene-on-copper piece so as to not fold in new wrinkles. **(C)** Placing amorphous holey carbon TEM grids on graphene-on-copper with amorphous carbon side of TEM grids touching the graphene. **(D)** Floating copper/graphene/TEM grids on sodium persulfate etchant. This removes the copper from the grids. **(E)** Graphene coated TEM grids after etching off copper. The solution is blue and there is no copper left on the graphene-coated grids. For size reference, the diameter of the glass Petri dish is approximately 6 cm and the glass slide is 7.5 cm by 2.5 cm. Please click here to view a larger version of this figure.

**Figure:**
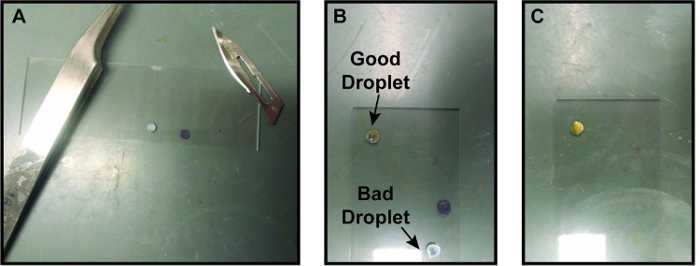

Figure 3**. Process for making graphene liquid cells (A)** Two graphene-coated TEM grids prepared on a glass slide with an edge cut off one of them. The surgical scalpel used to cut the grid is on the top right of the image. **(B)** Droplet of encapsulating solution on a graphene coated grid. The droplet on the top grid is the right size and has made a nice bead on the graphene. The droplet on the bottom grid has bled through the graphene, possibly due to a crack in the graphene. **(C)** Second graphene-coated grid placed on top of first grid with droplet of solution. This graphene liquid cell is now ready to load into a TEM. For size reference, the glass slide is 7.5 cm by 2.5 cm. Please click here to view a larger version of this figure.

**Figure:**
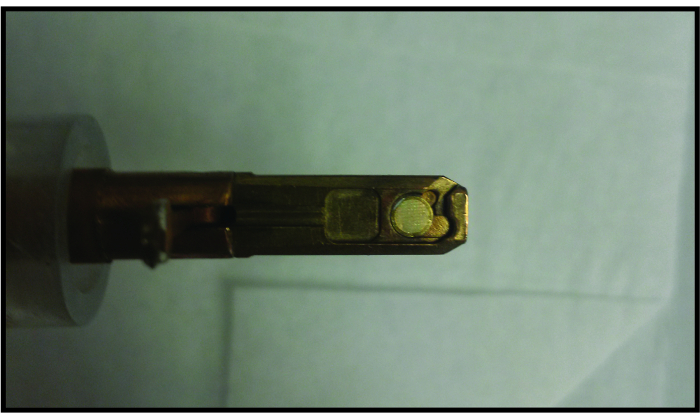

Figure 4**. Loading graphene liquid cell into standard single tilt TEM holder. **The graphene liquid cell fits in a standard single-tilt TEM holder in the same way a normal TEM grid fits in the holder. For size reference, the TEM grid has a diameter of 3 mm. Please click here to view a larger version of this figure.

**Figure:**
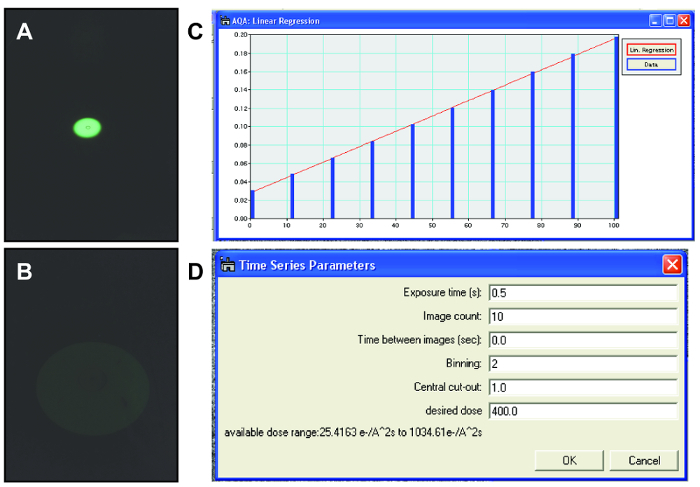

Figure 5**. TEM beam control. (A) **Condensed electron beam for dose rate calibration viewed using the fluorescent screen. **(B)** Expanded electron beam for dose rate calibration viewed using fluorescent screen. Intensity decreases as the electrons per area per time decrease which is why the electron beam is very faint. **(C) **Calibration curve relating the electron beam dose rate to the condenser lens current. This calibration curve is used for controlling the beam dose rate during imaging. **(D)** Parameters used when collecting TEM videos of nanoparticles in graphene liquid cells. Specific values used for each parameter may change depending on the material being imaged and the resolution needed. Please click here to view a larger version of this figure.

**Figure:**
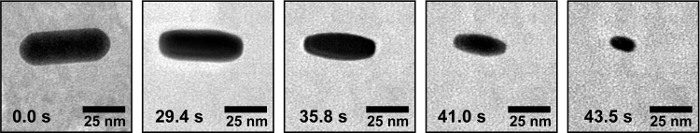

Figure 6**. Gold nanorod etching in a graphene liquid cell pocket.** Frames of a representative TEM video of a gold nanorod etching under dose rate of 800 e^-^/Å^2^s. After an initial period of no etching, the nanorod etches at a constant rate. Please click here to view a larger version of this figure.

**Figure:**
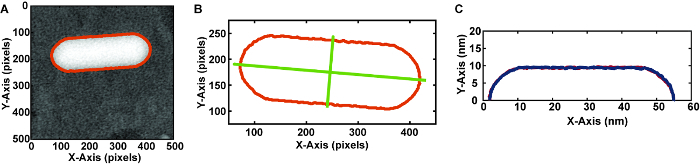

Figure 7**.Method for analyzing frames of video (A) **Outlining the nanorod using thresholding in image analysis software. (see **Table of Materials**) This separates the nanoparticle from the background and provides a shape for quantitative analysis. **(B)** Determining the major and minor axes of the nanorod. **(C)** Extracting each half of the 2-D outline cut along the major axis. Using these outlines, reconstruct the 3-D shape by rotating the outline around the x-axis. Please click here to view a larger version of this figure.

## Discussion

Graphene liquid cell electron microscopy can provide mechanistic information about nanocrystal growth and etching with high spatial resolution, but since making graphene liquid cells can be difficult and delicate, the technique requires attention to detail to extract usable data. Even after extensive practice making graphene liquid cells, only about a half to a quarter of made liquid cells successfully encapsulate the liquid solution. The critical step in forming liquid cells is placing the second grid on top of the droplet of liquid. Common errors include getting the tweezers stuck between the two grids, dropping the second grid too far off-center, and starting with a droplet that is too large. Since the assembly of graphene liquid cells is delicate and requires fine motor skills, it usually takes practice to successfully make the liquid pockets. Due to the expense of graphene-coated TEM grids, it is highly recommended that new graphene liquid cell users first practice the liquid cell making process on traditional copper, amorphous carbon TEM grids to save money.

Determining the causes of failure for liquid cells can be challenging because a researcher may not know if each step has been successful until imaging the sample at the end, and mistakes, like scratching the graphene, can go unnoticed. The easiest error to identify is an improper assembly because the researcher will immediately see liquid leaking out of the graphene liquid cell. Problems with making the graphene on copper grids, like cracking of the graphene, can be tougher to pinpoint. The quality of the graphene can be checked both before and after coating the TEM grids using Raman spectroscopy, but the graphene usually is unusable after this testing. Additionally, it is important to use direct transfer graphene because the two faces of graphene being put together need to be clean to properly form a seal through Van der Waals forces. Making graphene-coated grids through polymer transfer methods may leave polymer residue on the side of the graphene that is expected to bond together. If the correct procedure is followed using the correct TEM grids, lack of success with the graphene liquid cell is usually due to mishandling of the graphene and grids during assembly and fabrication.

Graphene liquid cell TEM advances existing liquid cell TEM techniques by using a much thinner encapsulation material that can used in any traditional TEM holder, making high resolution and facet trajectory tracking experiments much easier. With the resolution of commercial silicon nitride membrane liquid cells, much of the facet and kinetic information that can be attained by etching nanocrystals in the graphene liquid cell would be lost. Graphene liquid cell TEM experiments can also be performed on existing single tilt TEM holders negating the need for expensive new specialized holders. Further, the graphene liquid cell can be put in any holder that accepts standard TEM grid samples allowing for liquid cell experiments to be performed in advanced holders (heating, double tilt, cooling, cryo, cathodoluminescence) where silicon nitride liquid cells have not been designed. In addition, graphene liquid cells do not pose the risk of crashing the vacuum of the TEM column if the pockets rupture like other liquid cell TEM techniques. Although the graphene liquid cell is not a ubiquitous technique in nanocrystal fields yet, its ease of use and spatial resolution will make it much more widely used in the future.

Even with its many advantages, graphene liquid cell TEM does have limitations on the types of experiments that can be performed. Some liquid does evaporate as pockets form, so it is difficult to exactly determine the concentration of species in solution, even without considering electron beam effects. Graphene liquid cells also have random sizes, heights, and distributions of small pockets, so silicon nitride flow cells have the advantage of more quantifiable pre-beam concentrations and large, uniform liquid layers. As described in this work, only preloaded samples can be viewed using graphene liquid cell in the TEM, so it is not possible to flow in other solutions to trigger chemical reactions. The radiolysis species generated by the interaction of the electron beam with the liquid solution are the only trigger that can be used to start a reaction. Although not demonstrated yet, thermally initiated processes could be triggered in graphene liquid cells using standard heating holders. Electron beam-induced radiolysis effects are still not fully understood and can be difficult to control. Researchers have developed kinetic models to determine the contents of liquid cell pockets after beam interaction^31^,^32^, but their accuracy is limited by the number of reactions included in the model and any unknown concentration changes due to drying. Complex initial pocket contents with many reacting species like FeCl_3_, Tris Buffer, and even graphene^30^, can be difficult to fully understand using a kinetic model. Another disadvantage of liquid cell electron microscopy is that it is difficult to characterize the composition of the crystals formed during dynamic processes. For example, in growth experiments of multicomponent systems, it may be impossible to distinguish what phases or species are growing if the new nanocrystals are amorphous or not on zone axis. This is another reason why etching pre-formed nanocrystals of a known composition sitting on a known zone axis is desirable. Finally, there are still some arguments that beam-induced reactions in a graphene liquid cell do not represent the conditions of *ex situ* reactions in a flask.

Future graphene liquid cell experiments will help alleviate some of these concerns while also using new TEM advances to further probe the underlying mysteries of nanocrystals. Correlative *ex situ *nanocrystal synthesis and etching experiments will be critical in corroborating the mechanisms seen in liquid cell TEM experiments. Also, researchers have begun working on adding flow capabilities to graphene liquid cell TEM^35^ and making more controlled pockets^36^ including arrays of graphene liquid cells using lithographically prepared holes^37^. Advances in electron microscopy resolution and camera speed will make graphene liquid cell further able to study atomic dynamics during nanocrystal transformations. Wrapping small pockets of liquid in an atomically thin material like graphene for use in electron microscopy has a multitude of potential applications and will undoubtedly become a staple of nanoscience research in the future.

## Disclosures

The authors have nothing to disclose.
